# A Single Simulated Heliox Dive Modifies Endothelial Function in the Vascular Wall of ApoE Knockout Male Rats More Than Females

**DOI:** 10.3389/fphys.2019.01342

**Published:** 2019-10-22

**Authors:** Simin Berenji Ardestani, Vladimir V. Matchkov, Ingrid Eftedal, Michael Pedersen

**Affiliations:** ^1^Department of Clinical Medicine, Comparative Medicine Lab, Aarhus University, Aarhus, Denmark; ^2^Department of Circulation and Medical Imaging, Faculty of Medicine and Health Sciences, NTNU: Norwegian University of Science and Technology, Trondheim, Norway; ^3^Department of Biomedicine, Aarhus University, Aarhus, Denmark; ^4^Faculty of Nursing and Health Sciences, Nord University, Bodø, Norway

**Keywords:** endothelial dysfunction, apolipoprotein E, atherosclerosis, cardiovascular, saturation diving

## Abstract

**Introduction:**

The number of divers is rising every year, including an increasing number of aging persons with impaired endothelial function and concomitant atherosclerosis. While diving is an independent modulator of endothelial function, little is known about how diving affects already impaired endothelium. In this study, we questioned whether diving exposure leads to further damage of an already impaired endothelium.

**Methods:**

A total of 5 male and 5 female ApoE knockout (KO) rats were exposed to simulated diving to an absolute pressure of 600 kPa in heliox gas (80% helium, 20% oxygen) for 1 h in a dry pressure chamber. 10 ApoE KO rats (5 males, 5 females) and 8 male Sprague-Dawley rats served as controls. Endothelial function was examined *in vitro* by isometric myography of pulmonary and mesenteric arteries. Lipid peroxidation in blood plasma, heart and lung tissue was used as measures of oxidative stress. Expression and phosphorylation of endothelial NO synthase were quantified by Western blot.

**Results and Conclusion:**

A single simulated dive was found to induce endothelial dysfunction in the pulmonary arteries of ApoE KO rats, and this was more profound in male than female rats. Endothelial dysfunction in males was associated with changing in production or bioavailability of NO; while in female pulmonary arteries an imbalance in prostanoid signaling was observed. No effect of diving was found on mesenteric arteries from rats of either sex. Our findings suggest that changes in endothelial dysfunction were specific for pulmonary circulation. In future, human translation of these findings may suggest caution for divers who are elderly or have prior reduced endothelial function.

## Introduction

Diving is a popular physical activity, and the number of divers is increasing worldwide. In recent years, the average age of the recreational diving population has increased (Denoble et al., 2012; Berenji Ardestani et al., 2015). One-third of active United States scuba divers are reported >50 years old and exposed to several cardiovascular risk factors (Buzzacott et al., 2018). According to the International Marine Contractors Association, commercial divers at work must hold a valid certificate of medical fitness but no upper age limit is stated in the requirements. In the general population, endothelial dysfunction progresses with age and is associated with various cardiovascular diseases (Lakatta and Levy, 2003). Experimental and clinical evidence links endothelial dysfunction to oxidative stress, in which redox balances are disturbed by an imbalance of reactive oxygen species (ROS) and nitric oxide (NO) production (Cai and Harrison, 2000; El Assar et al., 2013; Higashi et al., 2014). In diving, excess oxidative stress is a prominent trait due to physical and chemical stress factors in the hyperbaric environment (Nossum et al., 1999, 2002; Brubakk et al., 2005; Obad et al., 2007; Eftedal et al., 2012, 2013; Mazur et al., 2014b). However, little is known about the effect of diving on the endothelial function in individuals who already are burdened with endothelial dysfunction.

Deficient apolipoprotein E (ApoE) expression impairs plasma lipoprotein metabolism and promotes the development of atherosclerosis (Davignon et al., 1988). ApoE knockout (KO) mice have frequently been used in studies of endothelial dysfunction associated with atherosclerosis and oxidative stress (Plump et al., 1992). These mice suffer from hypercholesterolemia even when they are fed a low-fat diet, and they develop atherosclerotic lesions in the aorta and large arteries already at 10 weeks of age (Plump et al., 1992). ApoE KO rats are now available and develop dyslipidemia and atherosclerotic plaques in carotid arteries already 12 weeks after onset of high-fat diet (Wei et al., 2015; Rune et al., 2018; Lee et al., 2019). This suggests endothelial dysfunction at early age of ApoE KO rats although this was not studied yet. In comparative studies that simulate human diving, rats have been used more than any other species (Lillo et al., 1985; Lillo and Parker, 2000; Bjorkum et al., 2017). In compressed gas diving, the pulmonary vasculature is exposed to oxidative stress and oxygen toxicity due to high partial pressure of oxygen and inert gas bubbles that develop during the decompression (ascent) phase (Butler and Hills, 1979; Wingelaar et al., 2017). Thus, pulmonary arteries may become susceptible to endothelial dysfunction mediated by diving procedures.

In this study, we hypothesized that simulated diving in arteriosclerosis-prone ApoE KO rats would cause endothelial dysfunction in pulmonary circulation, and that this deterioration would be larger in pulmonary than in peripheral, e.g., mesenteric, arteries (Mulvany and Aalkjaer, 1990). Since endothelial characteristics differ between the sexes, males and females were separately assessed in this study (Villar et al., 2008; Mazur et al., 2014a).

## Materials and Methods

Experiments were conducted in accordance with Animal Research: Reporting of *In Vivo* Experiments (ARRIVE) and the European Convention for the Protection of Vertebrate Animals used for experimental and other scientific purposes, and after permissions from the Norwegian ethical committee for animal experiments, approval number 16/210914; and Ministry of Environment and Food of Denmark, approval number 2018-15-0201-01477.

### Animals

A total of 10 male and 10 female ApoE KO rats (Horizon Discovery, Saint Louis, MO, United States) at the age of 6–9 weeks were used. This age can be considered as pre-atherosclerotic (Wei et al., 2015; Rune et al., 2018; Lee et al., 2019) with putative disturbance in endothelial function but no atherosclerotic plaques. All animals arrived to the facility at the age of 4 weeks old and were giving 2 weeks to acclimatize. 8 male Sprague-Dawley (SD) rats (210.1 ± 10.0 g) were included as controls. The ApoE KO rats are produced on the Sprague Dawley background (Rune et al., 2018). The animals were housed 5 per cage (temperature 21 ± 1°C, 12–12 h light-dark cycle) with *ad libitum* access to water and standard chow diet (Special diet service (SDS); Scanbur, Copenhagen, Denmark). The ApoE animals were randomly divided into diving and control groups; male diving (213.4 ± 18.9 g), male control (203.8 ± 23.9 g), female diving (178.2 ± 5.5 g) and female control (201.2 ± 9.5 g). Both diving and control animals were caged in the same cage in the weeks prior to experiment.

### Simulated Diving Protocol

**FIGURE 1:**
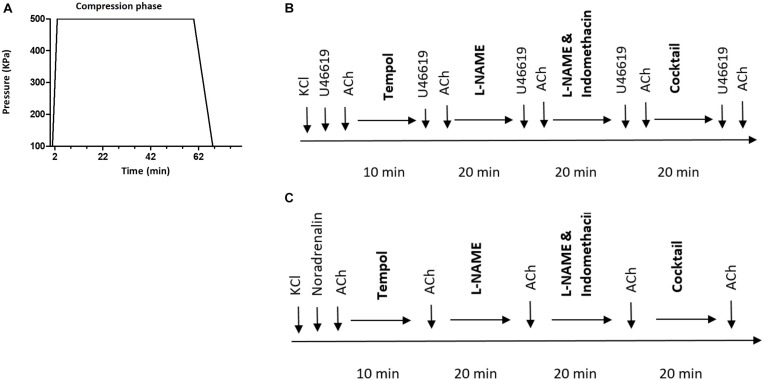
**(A)** Diving protocol. Each diving rat was exposed to hyperbaric heliox gas mixture (80% He and 20% O_2_) with compression rate of 200 kPa/min until it reached an absolute pressure of 600 kPa. The rat was exposed to hyperbaric conditions for 60 min followed by decompression at 50 kP/min; **(B)** schematic presentation of the protocol for myograph experiments on pulmonary and **(C)** mesenteric arteries. Cocktail, a mixture of L-NAME, indomethacin, Tram-34 and apamin; ACh, acetylcholine.

Each diving rat was exposed to a hyperbaric heliox gas mixture (80% He and 20% O_2_) in a 22 L hyperbaric chamber, starting at 8:00 am. The compression rate was 200 kPa/min until reaching an absolute pressure of 600 kPa, corresponding to 50 meters of seawater (msw). Conscious freely moving rats were exposed to the hyperbaric condition for 60 min, followed by a decompression stage to return to surface pressure at a decompression rate of 50 kP/min (Figure 1). Non-diving groups were not exposed to any sham diving.

### Post-diving Observation

After decompression, the animals were observed for 15 min to identify signs of decompression sickness, including abnormal walking, paralysis and twitching/convulsions (Pontier et al., 2009). Each rat was then anesthetized with a mixture of midazolam (0.5 mg/100 g), fentanyl (5 mg/100 g) and haldol (0.33 mg/100 g), euthanized by decapitation.

### Blood Sampling and Tissue Dissections

Blood samples from the right heart ventricle were collected immediately following anesthesia, in vacutainer 4 mL plastic lithium heparin tubes. The blood was centrifuged at 2200 g for 10 min at 20°C within 30 min of collection. Aspirated blood plasma was stored at −80°C until it was assayed. Heart ventricles and right lung were dissected and snap-frozen in liquid N_2_, before being stored at −80°C. The pulmonary artery (first bronchial artery in right lobe) and third order branch of the mesenteric artery were dissected. The arteries were transferred to cold physiological saline solution (PSS): NaCl, 119 mM; KCl, 4.7 mM; KH_2_PO4, 1.18 mM; MgSO4, 1.17 mM; NaHCO_3_, 25 mM; CaCl_2_, 1.6 mM; EDTA, 0.026 mM; and glucose, 5.5 mM, gassed with air, and pH adjusted to 7.4.

### Isometric Force Measurement

Immediately after dissection, each vessel was cleaned under a dissection microscope to remove surrounding connective tissues, cut into 2 mm pieces, and mounted in an isometric myograph (Danish Myo Technology, Aarhus, Denmark). The myograph chamber was filled with PSS, heated to 37°C, and constantly gassed with 5% CO_2_ in air. Force (in units of mN) was recorded with a PowerLab 4SP and Chart5 acquisition system (ADInstruments, Dunedin, New Zealand) and converted to wall tension (in units of N/m) by dividing the force with twice of vessel segment length.

After a 30 min equilibration period, the arteries were normalized to a passive wall tension where maximal contractile response was measured. This passive wall tension was equivalent to a lumen pressure of 3.9 kPa for the pulmonary artery (Nielsen et al., 2013) while the mesenteric artery segment was stretched to values corresponding 90% of the internal circumference of relaxed artery at 13.3 kPa (Mulvany et al., 1978). Maximal contractile response was assessed in the presence of 100 mM K^+^ ions in the bath solution (substituting Na^+^ ions with K^+^ ions in PSS). Contractility of pulmonary and mesenteric arteries were tested by cumulative applications of U46619, thromboxane analog (10^–8^ to 3 × 10^–6^ M) and noradrenaline (NA, 10^–8^ to 3 × 10^–5^ M), respectively. Endothelial function was assessed by relaxing pre-constricted arteries with acetylcholine (ACh: 10^–7^, 10^–6^ and 10^–5^ M). Pre-constrictions to approximately 80% of maximal constriction were obtained with either U46619 or NA-stimulations of the pulmonary and mesenteric arteries, respectively. Different components of the endothelium-dependent relaxation were inhibited by pre-incubation with inhibitors; arteries were pre-incubated for 20 min with non-selective inhibitor of NO synthase, 100 μM of N(G)-Nitro-*L*-arginine methyl-ester (L-NAME); with non-selective cyclooxygenase inhibitor, indomethacin (3 μM) and with a combination of small and intermittent Ca^2+^-activated K^+^ channel inhibitors, TRAM-34 (1 μM) and apamin (50nM), which have been shown to inhibit the endothelium-dependent hyperpolarizing factor (EDHF) response. Tempol (100 μM, 10 min pre-incubation) was used as superoxide scavenger. At the end of each experiment, endothelial-independent relaxation was tested by adding cumulative doses of sodium nitroprusside (SNP, 10^–8^–3 × 10^–5^ M). All drugs were purchased from Sigma-Aldrich (Oslo, Norway).

The experimental protocol is schematically shown in Figure 1. No time effect was observed in the separate time-control experiments (data not shown).

### Western Blot

Lung tissue was homogenized in lysis buffer (Tris–HCl 20 mM, ethylene glycol tetraacetic acid (5 mM), NaCl (150 mM), glycerophosphate (20 mM), NaF (10 mM), Triton X-100 (1%), Tween-20 (0.1%) and one tablet of protease inhibitor per 10 mL, pH adjusted to 7.5). 10 μg protein was loaded on gel (Criterion TGX gels 4–15%, cat #567-1085) and the gel was run for 1 h at 200 V, and then electrotransferred for 1 h at 100 V to nitrocellulose membranes. The membranes were blocked with 0.3% i-block in TBS-T and incubated with primary antibody overnight at 4°C, and, after washout, with horseradish-peroxidase (HRP)-conjugated secondary antibody (1:5000; Dako, Copenhagen, Denmark) for 2 h at room temperature. Excess antibody was removed by 4 times × 15 min washing, and bound antibody was detected by an enhanced chemiluminiscence kit (ECL, Amersham, United Kingdom). Protein amount was quantified using the ImageJ program (National Institutes of Health, Bethesda, MD, United States) (Bouzinova et al., 2014).

Different primary antibodies were used; e-NOS antibody (1:1.000; ab5589; Abcam, Cambridge, United Kingdom) and phospho-eNOS antibody (1:500; Ser1177; Cell Signaling Technology, Danvers, MA, United States). Pan-actin (1:1.000; #4968; Cell Signaling Technolog) served as loading reference.

### Lipid Peroxidation

Oxidative stress was evaluated by measuring the level of lipid peroxidation in heart and lung tissues. Formation of malondialdehyde (MDA) was measured using a thiobarbituric acid reactive substances (TBARS) kit (R&D System, Minneapolis, MN, United States). 10 mg of heart and lung tissue were lysed in 400 μl and 300 μl of lysis buffer (Tris–HCl 10 mM, sucrose 250 mM, EDTA 1 mM, EGTA 1 mM, Triton X-100 2%, pH adjusted to 7.4), respectively. Lung and heart lysates and plasma samples were prepared in accordance with the manufacturer’s protocol, and loaded onto a 96-well microplate. The plate was incubated for 2 h at 40–45°C, and MDA absorbance was measured at 532 nm (PHERAstar; BMG Labtech, Ortenberg, Germany). The results were calculated using the standard curve of TBARS Standard and normalized to the total protein content. Total protein content in blood plasma, lung and heart lysates was quantified by bicinchoninic acid (BCA) protein assay kit (Thermo Fisher Scientific, MA, United States) with loading 100 μl of working solution and 1 μl of sample to a 96-well microplate. Samples were measured at 562 nm (PHERAstar; BMG Labtech, Ortenberg, Germany) after 30 min of incubation at 37°C.

### Total Cholesterol

Total cholesterol was measured in blood plasma using cholesterol-reagents (Randox CH201; Randox Laboratories, Crumlin, United Kingdom). A volume of 1 μl plasma was mixed with 300 μl cholesterol reagents (1:100) in room temperature and transferred to a spectrophotometer 96-well microplate. Absorbance was measured at 500 nm and 37°C (PHERAstar; BMG Labtech, Ortenberg, Germany). The results were analyzed using a standard curve made with cholesterol standards and cholesterol reagent.

## Data Analyses

Vessel contraction was expressed relative to the maximal contraction of KCl (100% of contraction). Vessel relaxation was expressed in percentage of pre-constricted level (0% relaxation) to passive wall tension (100% relaxation). The effect of inhibitors was calculated as a comparison of difference in concentration-response curves before and after administration of the drug. Concentration–response curves were fitted to experimental data using four-parameter, non-linear regression curve fitting. From these curves, –logEC_50_, where EC_50_ was the concentration required to produce a half-maximal response, and maximal response were derived and compared using an extra sum-of-squares *F* test. Differences between means were tested by one-way ANOVA followed by Bonferroni *post hoc*-test or by *t*-test statistics. Results are presented as means ± SEM (standard error of the mean) for all analyses. Based on previous studies, a sample size of five rats per group was expected to give a power of 80%. *P* < 0.05 was considered statistically significant.

## Results

All diving rats completed the diving intervention without abnormal walking, paralysis or twitching/convulsions that would signify decompression sickness.

### Total Cholesterol Was Elevated in Blood Plasma of ApoE KO Rats

**FIGURE 2:**
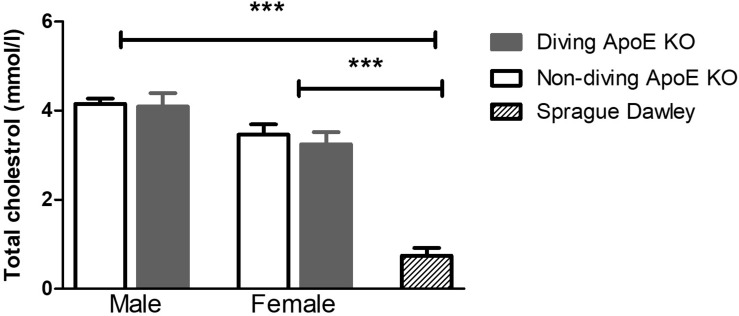
The total cholesterol in blood plasma from ApoE KO male and female rats was significantly higher than in Sprague Dawley rats that form a genetic background for ApoE KO rats, ^∗∗∗^*P* < 0.001, ANOVA, *n* = 5.

Plasma cholesterol level was significantly elevated in both male and female ApoE KO rats compared to normal plasma cholesterol in Sprague Dawley rats (Figure 2). There was no statistical difference in total plasma cholesterol between diving and non-diving groups, although there was a tendency for elevated cholesterol in males compared to females (Figure 2, *P* = 0.06).

### Pulmonary Arteries From Diving Rats Had Reduced no Production, While Only the Arteries From Female Non-diving Rats Had Increased Contribution of Pro-contractile Prostanoids

**FIGURE 3:**
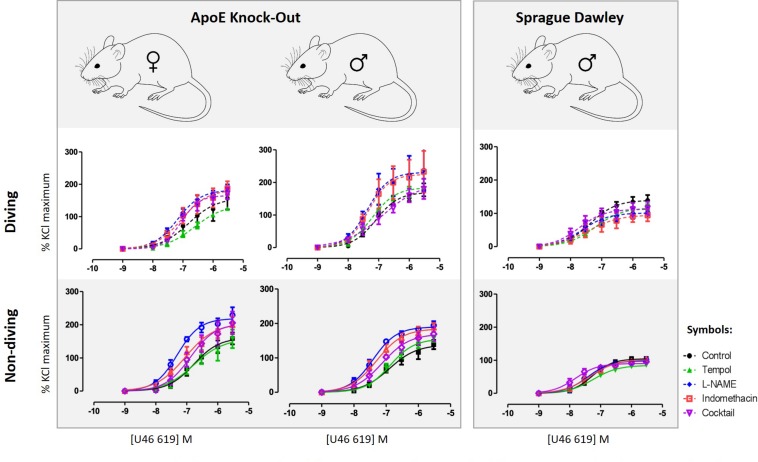
U46619 concentration-response curves of pulmonary arteries from different experimental groups under different experimental conditions; control conditions, following 10 min treatment with superoxide scavenger, Tempol (100 μM), incubation for 20 min with non-selective inhibitor of NO synthase, L-NAME (100 μM), incubation for 20 min with both L-NAME (100 μM) and indomethacin (3 μM); (e) 20 min incubation with L-NAME, indomethacin, TRAM-34 and apamin, i.e., pharmacological inhibition of all major pathways for endothelium-dependent relaxation. *F* test, *n* = 5.

Detailed are available in the Supplementary Figure S1. Contractile responses of pulmonary arteries to increasing concentrations of U46619 were compared between the experimental groups (Figure 3). There was no gender difference between contractile responses of pulmonary arteries from non-diving groups (Figure 3). A single diving simulation potentiated contractile responses under control conditions in pulmonary arteries from male, but not female rats (Figure 3). Diving significantly potentiated U46619 sensitivity of the arteries from male ApoE KO (Figure 3; -logEC_50_ was 7.08 ± 0.08 vs. 6.89 ± 0.14; *n* = 5, *P* < 0.001) and Sprague Dawley rats (Figure 3; -logEC_50_ was 7.36 ± 0.11 vs. 7.26 ± 0.08; *n* = 4, *P* < 0.001) in comparison with non-diving groups.

Incubation with tempol, a superoxide scavenger, had no effects on contractions of pulmonary arteries from male non-diving and diving ApoE KO rats (Figure 3). In the presence of tempol, pulmonary arteries from diving ApoE KO male still contracted stronger than non-diving ApoE KO controls (Figure 3; -logEC_50_ 7.20 ± 0.09 vs. 6.88 ± 0.07, respectively, *n* = 5, *P* < 0.001). In Sprague Dawley males, incubation with tempol significantly suppressed the contraction response in both diving and non-diving group (Figure 3; -logEC50 7.20 ± 0.12 vs. 7.23 ± 0.08, respectively, *n* = 4, *P* < 0.001). Contraction of pulmonary arteries from ApoE KO female rats was unaffected by tempol.

Incubation with L-NAME significantly potentiated contraction of pulmonary arteries from all experimental groups in ApoE rats (Figure 3; *P* < 0.01). In the presence of L-NAME, the difference between contractile responses of pulmonary arteries from male diving and non-diving ApoE KO was abolished (Figure 3; -logEC_50_, 7.32 ± 0.27 vs. 7.40 ± 0.06; *n* = 5, *P* = 0.69). Accordingly, L-NAME also potentiated the contraction of pulmonary arteries from non-diving female rats significantly stronger than the diving females (Figure 3; -logEC_50_, 7.30 ± 0.08 vs. 7.18 ± 0.08, *n* = 5, *P* < 0.0001). However, in Sprague Dawley males, incubation with L-NAME significantly suppressed the contraction response in diving pulmonary artery (Figure 3; -logEC50, 7.36 ± 0.11 vs. 7.61 ± 0.20, *n* = 4, *P* < 0.01).

Pulmonary artery contractile responses of diving and non-diving males were not changed by addition of indomethacin (Figure 3). Pre-incubation with L-NAME and indomethacin significantly suppressed contraction of female pulmonary arteries only from non-diving rats (Figure 3; -logEC_50_, 7.05 ± 0.17, *n* = 5, *P* < 0.01). After pharmacological inhibition of all major pathways for endothelium-dependent relaxation, i.e., pre-incubation with L-NAME, indomethacin, TRAM-34 and apamin, no difference between contractile responses of ApoE KO diving and non-diving males, and diving and non-diving females was found. The incubation potentiated the contractile response in male diving Sprague Dawley rats while significantly suppressed the response in ApoE KO male diving rats (Figure 3; -logEC50, 7.33 ± 0.02 vs. 6.84 ± 0.30, *n* = *5, P* < 0.02).

**FIGURE 4:**
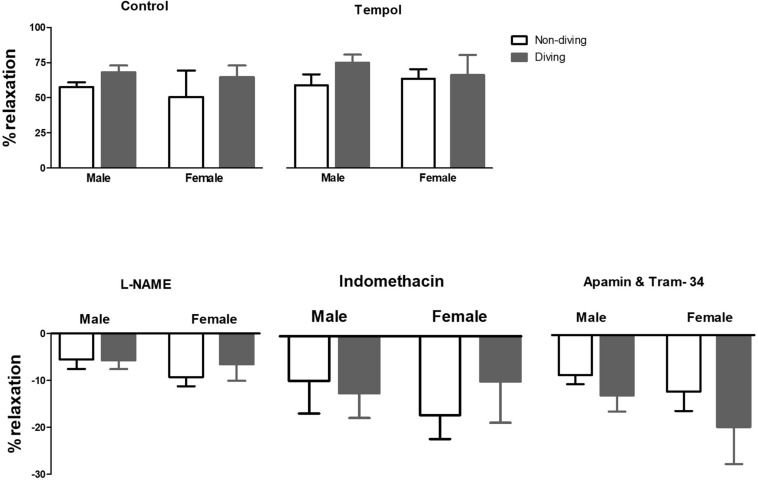
Percentage of relaxation in pulmonary arteries of ApoE KO rats in response to 10–5 M concentration of ACh in the presence of inhibitors for different endothelium-dependent relaxing pathways, ANOVA, *n* = 5.

**FIGURE 5:**
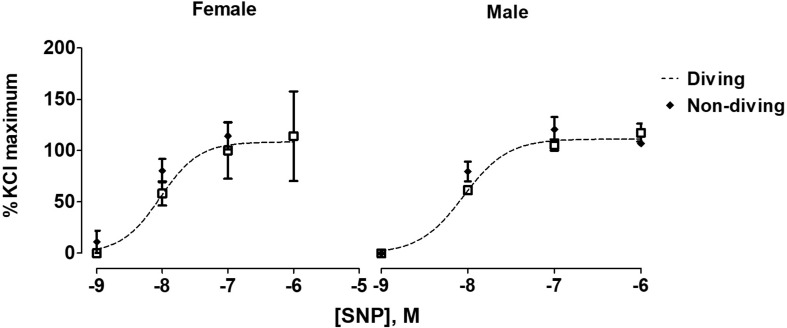
Concentration-response curves to endothelial-independent relaxation by SNP in pulmonary arteries of ApoE KO rats, *F* test.

When pre-constricted pulmonary arteries were compared for their ACh-induced relaxation responses, we found no difference between diving and non-diving rats (Figure 4). Accordingly, when endothelial-independent relaxation was tested by SNP, no difference between diving and non-diving groups or across sexes was found (Figure 5).

### No Differences Between Relaxation Responses of Mesentery Arteries to Increasing Concentrations of ACh

**FIGURE 6:**
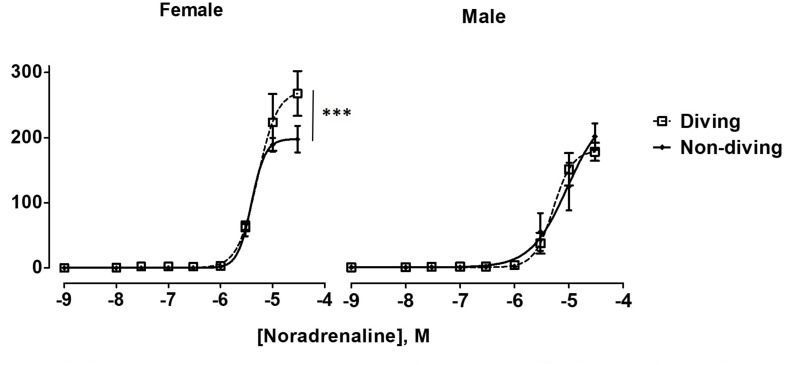
Noradrenaline concentration-response curves of mesentery arteries in ApoE KO rats from different experimental groups under control condition. To fit the experimental data to non-linear regression curves, noradrenaline concentration of 3 × 10^–5^ M was assumed to induce maximal contractile response in non-diving male rats. ^∗∗∗^*P* < 0.001, diving vs. non-diving female rats, *F* test, *n* = 5.

Maximal contractile responses to NA of mesenteric arteries from female diving rats were significantly larger in comparison with non-diving females and diving males (Figure 6; *P* < 0.001). There was no difference between diving and non-diving ApoE KO groups in NA-induced contraction of mesenteric small arteries from male rats (Figure 6; -logEC_50_ 5.28 ± 0.06 vs. 5.03 ± 0.2, *n* = 5).

**FIGURE 7:**
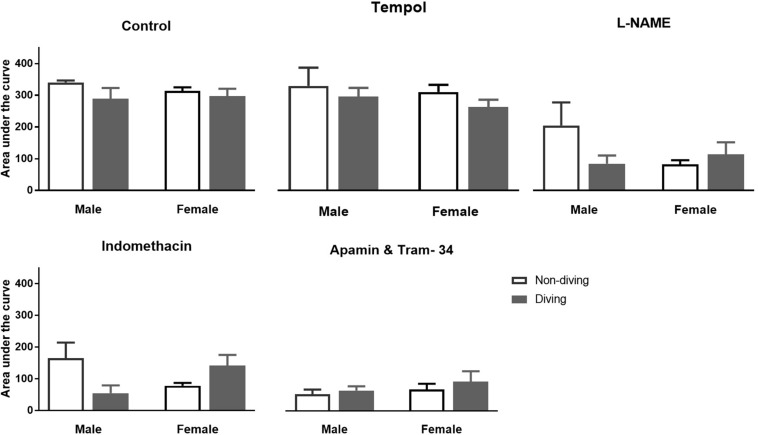
Area under curve for relaxation responses of mesenteric arteries in ApoE KO rats to acetylcholine (ACh) in the presence of inhibitors for different endothelium-dependent relaxing pathways; ANOVA and *t*-test.

**FIGURE 8:**
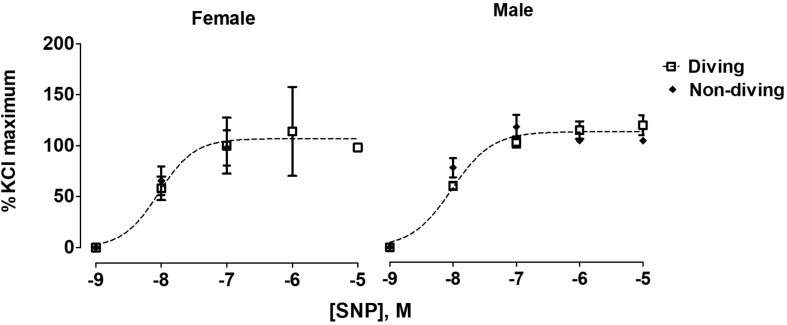
Concentration-response curves for endothelial-independent relaxations to SNP of mesentery arteries of ApoE KO rats from different experimental groups; *F* test, *n* = 5.

No differences between the experimental groups after pre-incubation with inhibitors for endothelium-dependent relaxing factors was observed (Figure 7). The endothelial-independent relaxation in response to SNP was similar in mesenteric arteries from all experimental groups (Figure 8).

### No Association Between Diving Simulation and Phosphorylation of eNOS and Oxidative Stress

**FIGURE 9:**
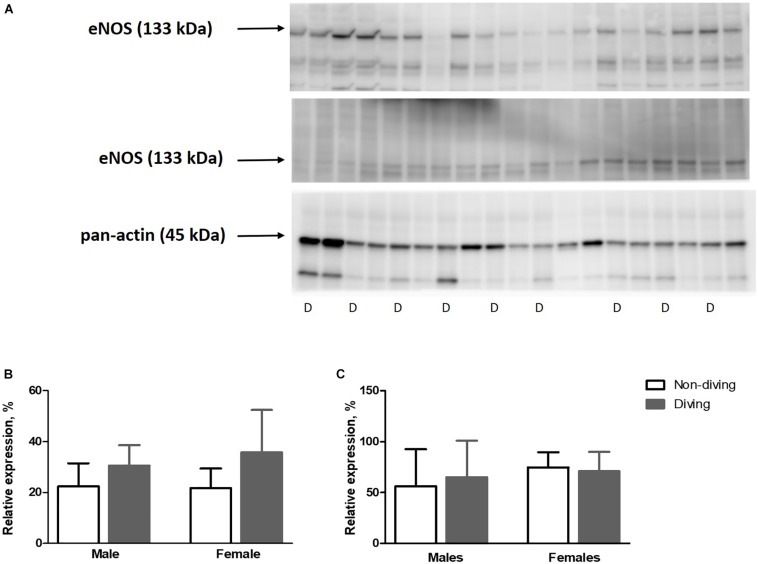
**(A)** Relative expression of eNOS and p-eNOS proteins quantified by Western blot and normalized to pan-actin as housekeeping protein ApoE KO rats, **(B)**, relative expression of eNOS normalized to pan-actin **(C)**, p-eNOS/eNOS in lung tissue, *n* = 5. eNOS, endothelial nitric oxide synthase; p-eNOS, phosphorylated eNOS; D, diving; ANOVA and *t*-test.

Our functional study suggested that diving affected NO production in the pulmonary artery of ApoE KO rats. This finding might be due to either changes in eNOS expression, changes in its activation by phosphorylation or NO scavenging in the vascular wall. However, we found no difference in eNOS expression between the groups (Figure 9). The relative amount of phosphorylated eNOS (p-eNOS) tended toward an increase in the male diving group (Figure 9; 56.17 ± 36.57 vs. 65.08 ± 35.84, *n* = 5), suggesting that reduced NO production was not the reason for observed changes in the vascular tone of pulmonary arteries from diving male rats.

**FIGURE 10:**
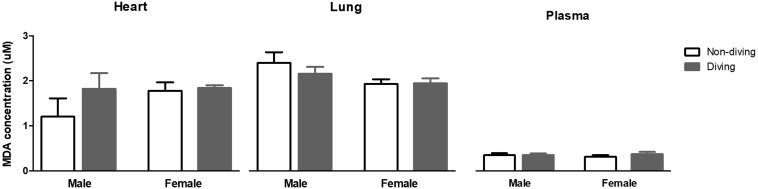
Lipid peroxidation in blood plasma, lung and heart measured as malondialdehyde (MDA) concentration, and normalized to total protein content in ApoE KO rats; ANOVA and *t*-test.

Free radicals might scavenge NO in the vascular wall, and for this reason, we evaluated oxidative stress by measuring lipid peroxidation level in blood plasma, lung and heart tissues. However, no differences were observed between the groups (Figure 10).

## Discussion

**FIGURE 11:**
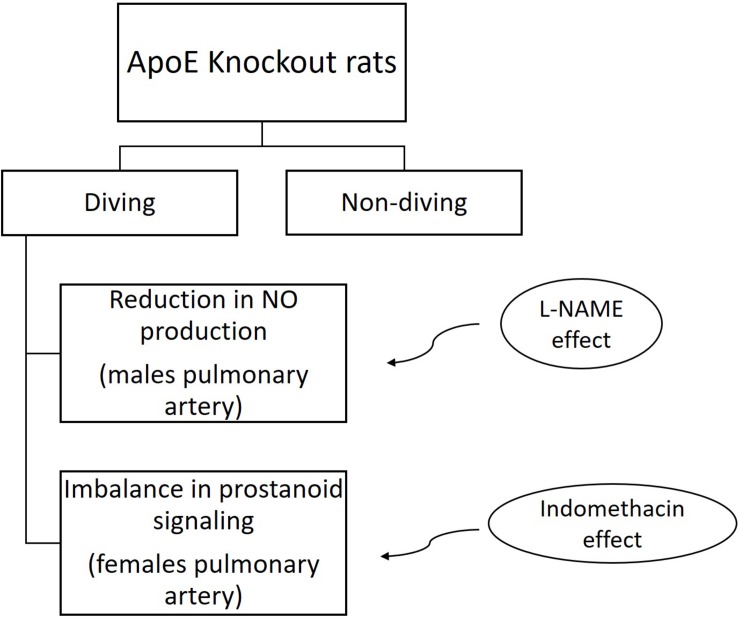
Summery chart of main results.

The main finding of this study was that simulated diving caused endothelial dysfunction in the pulmonary arteries of ApoE KO rats. In mesenteric arteries from female rats, the contractile response to NA was potentiated after a single diving simulation but does not confirm any endothelial dysfunction since no difference after pre-incubation with endothelium-dependent relaxation inhibitors was observed. This is the first study to include rats that are genetically prone to atherosclerosis, and it supports previous reports on both humans and animals (Nossum et al., 1999; Brubakk et al., 2005). Interestingly, we found that endothelial dysfunction after diving was more severe in male than in female ApoE KO rats. Endothelial dysfunction in males’ pulmonary arteries was associated with changing in production or bioavailability of NO. We also observed an imbalance in prostanoid signaling in female pulmonary arteries (Figure 11).

Previous studies on humans and animal models have shown that diving causes endothelial dysfunction. An early study on rabbits exposed to simulated diving suggested that endothelial dysfunction in the pulmonary artery arose from mechanical disruption caused by decompression-induced vascular bubbles (Nossum et al., 1999). Later, the endothelial dysfunction and reduction of flow-mediated dilatation (FMD) in the human brachial artery was reported after SCUBA dives with few bubbles (Brubakk et al., 2005). SCUBA diving has since then been shown to affect endothelium-dependent relaxation responses in micro and macro vasculature (Lambrechts et al., 2013). Accordingly, an impaired contractile response to phenylephrine in aorta and mesentery arteries from male rats has been reported as a result of vascular smooth muscle injury without any changes in endothelium-dependent relaxation (Mazur et al., 2014b, 2016). In the present study, we also found no difference in ACh-induced relaxations in neither pulmonary or mesentery arteries. However, the U46619 induced contraction was potentiated in male ApoE KO diving rats compared to the non-diving groups, which is contrary to previous *ex vivo* studies (Mazur et al., 2016). We observed similar results in the Sprague Dawley diving rat suggesting that these changes were due to the diving exposure itself and not the nature of ApoE KOs.

ApoE plays a major physiological role in lipoprotein metabolism; ApoE deficiency is associated with hypercholesterolemia. This makes ApoE KO animals appropriate models for studies of atherosclerotic diseases, which are usually absent in rodents (Meir and Leitersdorf, 2004; Rune et al., 2018; Lee et al., 2019). In present study, the rats were fed a chow diet, allowing investigations of the endothelial function at early stage of atherosclerosis and before development of atherosclerotic lesions (d’Uscio et al., 2001; Van Assche et al., 2007). However, ApoE KO rats still had remarkably higher amounts of total cholesterol compared to the Sprague Dawley rats that form the genetic background for the ApoE KOs. Hypercholesterolemia triggers oxidative stress due to increased production of O_2_^–^ and degradation and/or inactivation of NO, leading to endothelium-dependent relaxation dysfunction in ApoE KO animals (Rosenfeld et al., 2002). Importantly, in cardiovascular disease, there is often imbalance between endothelium-derived relaxing factors and endothelium-derived contracting factors, and this shift is usually in favor of the endothelium-derived contracting factors (Gluais et al., 2005; Félétou et al., 2011). This finding could explain the differences between the non-diving ApoE KO and Sprague Dawley rats in this study.

We should emphasize that while we chose to study the pulmonary artery, most of previous *ex vivo* diving studies have examined mesenteric artery and/or aorta. This could explain some of the differences between the current and previously reported results. Due to low pulmonary resistance the pressure in pulmonary artery is much lower than in the systemic arteries. In compressed gas diving, the pulmonary vasculature is exposed to high partial pressure of oxygen levels that border on oxygen toxicity, and causing high levels of oxidative stress (Butler and Hills, 1979; Malik, 2016; Wingelaar et al., 2017). Therefore, we consider the pulmonary artery to be highly relevant in diving studies. We assessed oxidative stress by both MDA production measurement and as a contraction to U46619 in the presence of antioxidant tempol. However, we found no indication of increased ROS production that could explain the differences between diving and non-diving rats. It may be worth noting that ventilation with helium has been reported to have an anti-inflammatory effect (Brubakk et al., 2014; Rocco et al., 2019), which may have reduced any side effect of potential O_2_ toxicity.

We found no difference in ACh responses in the pulmonary arteries between diving or non-diving groups in this study. This could however be due to technical issues. Pulmonary arteries show tachyphylaxis when accumulatively stimulated with increasing concentrations of ACh. We chose therefore to test the relaxation of pulmonary arteries with applying a single dose of ACh (Boedtkjer et al., 2011). The endothelium-independent relaxation was tested by SNP and found to be unaffected in any groups, in agreement with a previous report (Mazur et al., 2014b). The incubation with L-NAME caused the differences between ApoE KO diving/non-diving and Sprague Dawley rats in U46619 induced contraction. The contraction was potentiated in ApoE KO rats while it was suppressed in Sprague Dawley. L-NAME is a non-selective NO inhibitor, and the observed differences between diving and non-diving ApoE KO rats could be due to changing in production or bioavailability of NO (Okon et al., 2003). However, the mechanism behind suppression of contraction by L-NAME in Sprague Dawley rats is surprising but this could be some indirect effect as it was previously been shown for inhibiting effect of L-NAME on hypoxia-induced contraction (Terraz et al., 1999). As mentioned above, due to hypercholesterolemia, NO production was impaired in APoE KOs compared to the Sprague Dawley rats (Rosenfeld et al., 2002), and we hypothesized that it was even further damaged in diving ApoE KOs. We did not observe any difference in contractile responses to U46619 in pulmonary arteries after inhibition of all endothelium-dependent relaxation factors suggesting that smooth muscle cell function was not affected.

The diving female ApoE KO rats’ pulmonary arteries were not affected by L-NAME incubation. U46619 induced contraction was significantly suppressed following incubation with both L-NAME and indomethacin which could be a consequence of changes in prostanoid signaling. We suggest that female APoE KO rats were not affected by diving but this is not surprising; previous studies have shown that endothelium-dependent relaxation is more severely impaired in atherosclerosis arteries of males compared to females, both in humans and animals (Bossaller et al., 1987; Bonthu et al., 1997).

## Conclusion

In summary, endothelial dysfunction after a single dry heliox dive was associated with changing in bioavailability of NO in males, and altered prostanoid signaling in female ApoE KO rats. Whether repeated diving over time causes persistent changes in endothelial function in divers with atherosclerosis, either worsening from excess oxidative stress or improving via acclimatization, should be evaluated in future studies.

## Limitations

In this study, we focused on the function of the vascular endothelium. Since the changes in vascular smooth muscle cell function after simulated diving were previously reported (Mazur et al., 2014b) we cannot rule out that this may also been the case in this study. We did not assess decompression-induced microbubbles as we prioritized measuring endothelial function as soon as possible without exposure to prolonged anesthesia, which is necessary for ultrasound imaging. Future studies could benefit from the assessment of bubble formation.

## Data Availability Statement

All datasets generated for this study are included in the manuscript/Supplementary Files.

## Ethics Statement

The animal study was reviewed and approved by Norwegian ethical committee for animal experiments, approval number 16/210914 and Ministry of Environment and Food of Denmark, approval number 2018-15-0201-01477.

## Author Contributions

All authors designed the study. SB managed the data collection, laboratory work and analysis, and drafted the manuscript. SB and VM conducted the statistical analysis. All the co-authors contributed in the final correction and writing.

## Conflict of Interest

The authors declare that the research was conducted in the absence of any commercial or financial relationships that could be construed as a potential conflict of interest. The reviewer AM declared a past co-authorship with one of the authors VM.
