# Supplementary figures and images for: A Single Simulated Heliox Dive Modifies Endothelial Function in the Vascular Wall of ApoE Knockout Male Rats More Than Females
Source: Front Physiol. 2019 Oct 22;10:1342. doi: 10.3389/fphys.2019.01342 (PMC6817487; doi:10.3389/fphys.2019.01342)

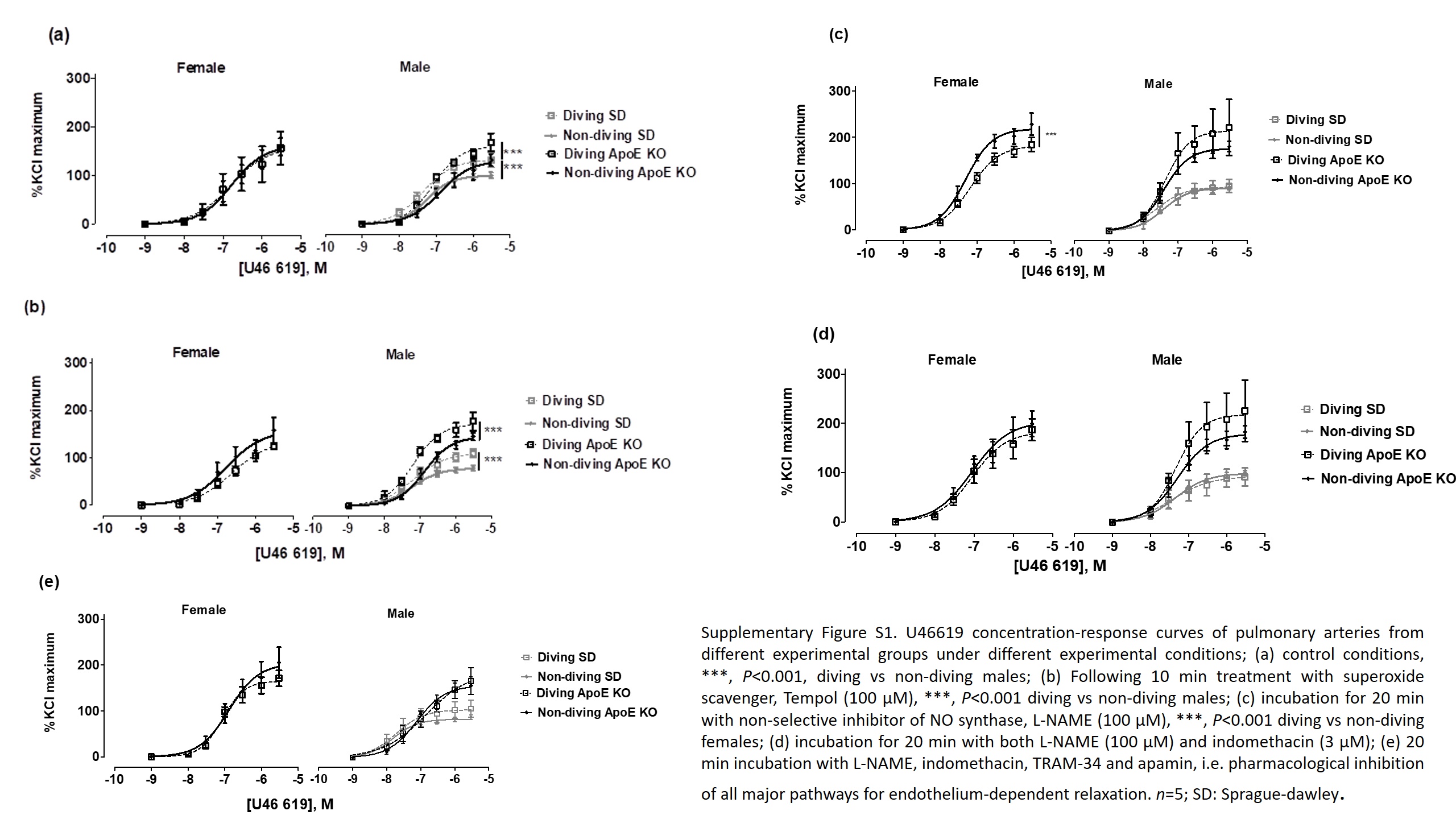

Supplement: Supplementary file 1 [file Image_1.jpg]
